# 1,1-Bis[4-(trifluoro­meth­yl)phen­yl]germetane

**DOI:** 10.1107/S1600536809015268

**Published:** 2009-04-30

**Authors:** Lawrence A. Huck, Gregory D. Potter, Hilary A. Jenkins, James F. Britten, William J. Leigh

**Affiliations:** a Department of Chemistry, McMaster University, Hamilton, ON, Canada L8S 4M1

## Abstract

The inter­nal C—Ge—C bond angle in the germacyclo­butane ring of the title compound, C_17_H_14_F_6_Ge or [Ge(C_3_H_6_)(C_7_H_4_F_3_)_2_], is 77.8 (3)°. The –CF_3_ groups display rotational disorder [occupancies 0.604 (14):0.396 (14) and 0.410 (6):0.411 (6):0.179 (3)] and the germacyclo­butane ring also shows disorder [occupancies 0.604 (14):0.396 (14)].

## Related literature

For the synthesis of the title compound, see: Leigh *et al.* (2008 ▶). For related compounds see: Tokitoh *et al.* (1995 ▶); Eichler *et al.* (1999 ▶); Meiners *et al.* (2002 ▶); Tajima *et al.* (2005 ▶). For 1,1-bis[3,5-bis(trifluoromethyl)phenyl]germetane, which exhibits similar structural features, see: Potter *et al.* (2009 ▶).
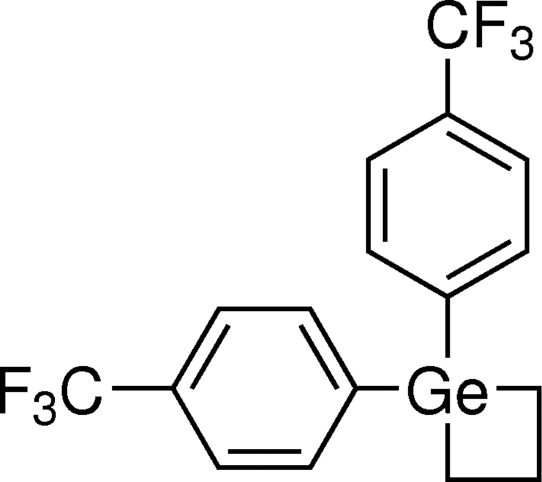


## Experimental

### 

#### Crystal data

[Ge(C_3_H_6_)(C_7_H_4_F_3_)_2_]
*M* *_r_* = 404.87
Monoclinic, 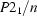

*a* = 13.596 (6) Å
*b* = 6.412 (3) Å
*c* = 20.243 (9) Å
β = 107.081 (7)°
*V* = 1687 (1) Å^3^
*Z* = 4
Mo *K*α radiation
μ = 1.87 mm^−1^
*T* = 173 K
0.40 × 0.38 × 0.20 mm

#### Data collection

Bruker SMART CCD area-detector diffractometer
Absorption correction: multi-scan *SADABS* (Bruker, 1997 ▶) *T*_min_ = 0.484, *T*_max_ = 0.688
14478 measured reflections
3867 independent reflections
2378 reflections with *I* > 2σ(*I*)
*R*_int_ = 0.053

#### Refinement

*R*[*F* ^2^ > 2σ(*F* ^2^)] = 0.041
*wR*(*F* ^2^) = 0.097
*S* = 0.99
3867 reflections
258 parameters
19 restraints
H-atom parameters constrained
Δρ_max_ = 0.68 e Å^−3^
Δρ_min_ = −0.61 e Å^−3^

### 

Data collection: *SMART* (Bruker, 1997 ▶); cell refinement: *SAINT* (Bruker, 1997 ▶); data reduction: *SAINT*; program(s) used to solve structure: *SHELXS97* (Sheldrick, 2008 ▶); program(s) used to refine structure: *SHELXL97* (Sheldrick, 2008 ▶); molecular graphics: *SHELXTL* (Sheldrick, 2008 ▶); software used to prepare material for publication: *SHELXTL*.

## Supplementary Material

Crystal structure: contains datablocks I, global. DOI: 10.1107/S1600536809015268/im2108sup1.cif

Structure factors: contains datablocks I. DOI: 10.1107/S1600536809015268/im2108Isup2.hkl

Additional supplementary materials:  crystallographic information; 3D view; checkCIF report

## Figures and Tables

**Fig. 1.**
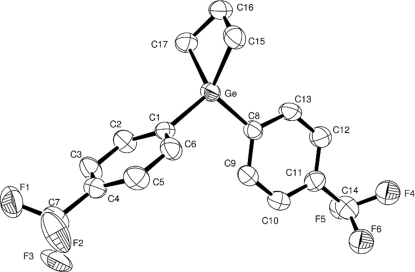
The molecular structures of (I) drawn with 50% probability ellipsoids. H atoms not shown. Both trifluoromethyl groups and the (CH2)3 group are disordered but the disorder is not shown for clarity.

**Fig. 2.**
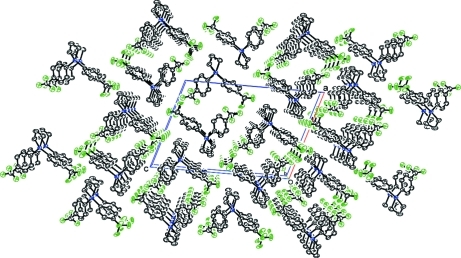
Packing diagram of (I) in the space group P 21/n.

## supplementary crystallographic information

### Comment

Photolysis of 1,1-diarylgermetanes results in two competing modes of cycloreversion: (2 + 2) to yield the corresponding 1,1-diarylgermene (*R*_2_Ge=CH_2_) and ethylene, and (3 + 1) to yield the corresponding diarylgermylene (*R*_2_Ge) and cyclopropane (Leigh *et al.*, 2008). While the quantum yield for decomposition of the germetane remains roughly constant regardless of aromatic substitutent (Φ*ca* 0.10), decreasing the electron density at germanium by aromatic ring substitution favors formation of the germylene and cyclopropane (Leigh *et al.*, 2008). The molecular structure of (I) is shown in Figure 1. The internal C—Ge—C bond angle is 77.8 (3)°.

To our knowledge, there are no other reported crystal structures of germacyclobut*anes* (*i.e.* those in which the three carbon atoms of the four membered ring are all saturated) with which to compare these data. There are, however, several reported crystal structures of germacyclobut*enes* to which limited comparisons can be made (Tokitoh *et al.*, 1995; Eichler *et al.*, 1999; Meiners *et al.*, 2002; Tajima *et al.*, 2005). The endocyclic Ge—C bond distances are quite similar, despite the carbon-carbon double bond within the ring, and the endocyclic C—Ge—C angle is slightly smaller in these germacyclobutene molecules, which is to be expected. The 4-membered germacyclobutane ring of the title compound is not planar. The angle between the plane made by the carbon atoms in the ring (C15—C17) and the plane containing the germanium and two adjacent carbon atoms in the ring (C15, C17) is 24 (2)°. The aromatic rings are nearly perpendicular, with an angle of 85.8 (1)° between them.

The trifluoromethyl groups are disordered and have been refined as such (see refinement details). The two CF_3_ groups interact with each other and the germacyclobutane ring of neighbouring molecules (see the packing diagram - Figure 2). The non-planarity of the germacyclobutane ring results in disorder that has been refined over two occupancy sites.

The crystal structure of an analogue of (I) in which the aromatic rings are substituted at the 3 and 5 positions with CF_3_ groups [*i.e*. 1,1-bis(3,5-bis(trifluoromethyl)phenyl)germetane] exhibits similar structural features (Potter, *et al.*, 2009).

### Experimental

Compound (I) was synthesized as described elsewhere (Leigh *et al.*, 2008). Single crystals for *x*-ray diffraction were obtained *via* repeated recrystallizations from methanol.

### Refinement

The data were collected at -100°C on a single-crystal mounted in a cryoloop. X-ray crystallographic analysis was performed at the McMaster Analytical X-Ray (MAX) Diffraction Facility. Hydrogen atoms were treated as riding upon their parent atoms with C—H distances of 0.95 Å (aromatic) and 0.96 Å (CH_2_) and with *U*_iso_(H) = 1.2*U*_eq_(C). Fluorine atoms at C7/C7A were split over two positions, with approximately 60:40 occupancy. Fluorine atoms at C14/C14A/C14' were disordered over three positions (41:41:18 occupancy) with one thermal parameter (0.06). The propyl moiety of the germacycle is disordered over two sites (60:40 occupancy) and a restraint is applied so that the groups can twist away from each other but not leave the sphere of germanium.

### Crystal data

**Table:**

[Ge(C_3_H_6_)(C_7_H_4_F_3_)_2_]	*F*(000) = 808
*M**_r_* = 404.87	*D*_x_ = 1.594 Mg m^−^^3^
Monoclinic, *P*2_1_/*n*	Melting point: 319 K
Hall symbol: -P 2yn	Mo *K*α radiation, λ = 0.71073 Å
*a* = 13.596 (6) Å	Cell parameters from 5916 reflections
*b* = 6.412 (3) Å	θ = 3.5–26.6°
*c* = 20.243 (9) Å	µ = 1.87 mm^−^^1^
β = 107.081 (7)°	*T* = 173 K
*V* = 1687 (1) Å^3^	Block, colourless
*Z* = 4	0.40 × 0.38 × 0.20 mm

### Data collection

**Table:**

Bruker SMART CCD area-detector diffractometer	3867 independent reflections
Radiation source: fine-focus sealed tube	2378 reflections with *I* > 2σ(*I*)
graphite	*R*_int_ = 0.053
φ and ω scans	θ_max_ = 27.5°, θ_min_ = 1.6°
Absorption correction: multi-scan *SADABS*	*h* = −17→17
*T*_min_ = 0.484, *T*_max_ = 0.688	*k* = −8→8
14478 measured reflections	*l* = −26→25

### Refinement

**Table:**

Refinement on *F*^2^	Primary atom site location: structure-invariant direct methods
Least-squares matrix: full	Secondary atom site location: difference Fourier map
*R*[*F*^2^ > 2σ(*F*^2^)] = 0.041	Hydrogen site location: inferred from neighbouring sites
*wR*(*F*^2^) = 0.097	H-atom parameters constrained
*S* = 0.99	*w* = 1/[σ^2^(*F*_o_^2^) + (0.0275*P*)^2^ + 2.3419*P*] where *P* = (*F*_o_^2^ + 2*F*_c_^2^)/3
3867 reflections	(Δ/σ)_max_ = 0.022
258 parameters	Δρ_max_ = 0.68 e Å^−^^3^
19 restraints	Δρ_min_ = −0.61 e Å^−^^3^

### Special details

**Table:**

Geometry. All e.s.d.'s (except the e.s.d. in the dihedral angle between two l.s. planes) are estimated using the full covariance matrix. The cell e.s.d.'s are taken into account individually in the estimation of e.s.d.'s in distances, angles and torsion angles; correlations between e.s.d.'s in cell parameters are only used when they are defined by crystal symmetry. An approximate (isotropic) treatment of cell e.s.d.'s is used for estimating e.s.d.'s involving l.s. planes.
Refinement. Refinement of *F*^2^ against ALL reflections. The weighted *R*-factor *wR* and goodness of fit *S* are based on *F*^2^, conventional *R*-factors *R* are based on *F*, with *F* set to zero for negative *F*^2^. The threshold expression of *F*^2^ > σ(*F*^2^) is used only for calculating *R*-factors(gt) *etc*. and is not relevant to the choice of reflections for refinement. *R*-factors based on *F*^2^ are statistically about twice as large as those based on *F*, and *R*- factors based on ALL data will be even larger.

### Fractional atomic coordinates and isotropic or equivalent isotropic displacement parameters (Å^2^)

**Table:**

	*x*	*y*	*z*	*U*_iso_*/*U*_eq_	Occ. (<1)
Ge1A	0.88719 (3)	0.20119 (6)	0.19017 (2)	0.03626 (13)	0.396 (14)
C15A	0.7582 (7)	0.347 (4)	0.1861 (18)	0.049 (3)	0.396 (14)
H15C	0.7483	0.3738	0.2319	0.058*	0.396 (14)
H15D	0.7466	0.4755	0.1576	0.058*	0.396 (14)
C16A	0.6994 (5)	0.152 (3)	0.1482 (9)	0.058 (3)	0.396 (14)
H16C	0.6402	0.1931	0.1086	0.070*	0.396 (14)
H16D	0.6745	0.0638	0.1801	0.070*	0.396 (14)
C17A	0.7845 (7)	0.034 (4)	0.1227 (15)	0.0493 (15)	0.396 (14)
H17C	0.7811	0.0624	0.0740	0.059*	0.396 (14)
H17D	0.7889	−0.1177	0.1325	0.059*	0.396 (14)
Ge1	0.88719 (3)	0.20119 (6)	0.19017 (2)	0.03626 (13)	0.604 (14)
C15	0.7486 (5)	0.302 (3)	0.1857 (11)	0.049 (3)	0.604 (14)
H15A	0.7232	0.2525	0.2240	0.058*	0.604 (14)
H15B	0.7400	0.4551	0.1796	0.058*	0.604 (14)
C16	0.7073 (5)	0.1749 (18)	0.1173 (6)	0.058 (3)	0.604 (14)
H16A	0.6958	0.2660	0.0763	0.070*	0.604 (14)
H16B	0.6424	0.1022	0.1156	0.070*	0.604 (14)
C17	0.7979 (11)	0.013 (6)	0.122 (2)	0.0493 (15)	0.604 (14)
H17A	0.8172	−0.0008	0.0786	0.059*	0.604 (14)
H17B	0.7874	−0.1249	0.1407	0.059*	0.604 (14)
C1	0.9675 (3)	0.0705 (5)	0.27568 (18)	0.0341 (8)	
C2	1.0113 (3)	−0.1241 (6)	0.2752 (2)	0.0445 (9)	
H2A	1.0030	−0.1936	0.2324	0.053*	
C3	1.0670 (3)	−0.2189 (6)	0.3359 (2)	0.0485 (10)	
H3A	1.0965	−0.3526	0.3346	0.058*	
C4	1.0799 (3)	−0.1212 (6)	0.39821 (19)	0.0407 (9)	
C5	1.0369 (3)	0.0734 (6)	0.3998 (2)	0.0471 (10)	
H5A	1.0456	0.1424	0.4427	0.056*	
C6	0.9812 (3)	0.1671 (5)	0.3389 (2)	0.0444 (9)	
H6A	0.9517	0.3006	0.3404	0.053*	
C8	0.9769 (3)	0.3844 (5)	0.15718 (18)	0.0379 (8)	
C9	1.0823 (3)	0.3923 (6)	0.1889 (2)	0.0470 (10)	
H9A	1.1126	0.3007	0.2262	0.056*	
C10	1.1437 (3)	0.5314 (6)	0.1669 (2)	0.0510 (10)	
H10A	1.2158	0.5345	0.1889	0.061*	
C11	1.1000 (3)	0.6659 (6)	0.1130 (2)	0.0448 (9)	
C12	0.9955 (3)	0.6588 (6)	0.0806 (2)	0.0512 (10)	
H12A	0.9653	0.7506	0.0433	0.061*	
C13	0.9352 (3)	0.5183 (6)	0.1024 (2)	0.0471 (10)	
H13A	0.8635	0.5130	0.0795	0.057*	
C7	1.1400 (4)	−0.2268 (7)	0.4628 (2)	0.0561 (11)	0.604 (14)
F1	1.1175 (11)	−0.4307 (12)	0.4626 (7)	0.083 (3)	0.604 (14)
F2	1.1343 (13)	−0.146 (2)	0.5219 (6)	0.078 (4)	0.604 (14)
F3	1.2396 (5)	−0.227 (3)	0.4671 (8)	0.072 (3)	0.604 (14)
C7A	1.1400 (4)	−0.2268 (7)	0.4628 (2)	0.0561 (11)	0.396 (14)
F1A	1.0871 (16)	−0.380 (3)	0.4803 (12)	0.106 (7)	0.396 (14)
F2A	1.1506 (18)	−0.091 (3)	0.5158 (8)	0.070 (5)	0.396 (14)
F3A	1.2337 (11)	−0.281 (4)	0.4667 (12)	0.091 (8)	0.396 (14)
C14	1.1663 (3)	0.8216 (7)	0.0908 (2)	0.0589 (11)	0.410 (6)
F4	1.1154 (5)	1.0064 (10)	0.0742 (5)	0.0613 (7)*	0.410 (6)
F5	1.1939 (6)	0.7630 (11)	0.0372 (4)	0.0613 (7)*	0.410 (6)
F6	1.2236 (6)	0.9328 (12)	0.1444 (3)	0.0613 (7)*	0.410 (6)
C14'	1.1663 (3)	0.8216 (7)	0.0908 (2)	0.0589 (11)	0.411 (6)
F4'	1.1110 (5)	0.9575 (12)	0.0424 (5)	0.0613 (7)*	0.411 (6)
F5'	1.2282 (6)	0.7196 (11)	0.0569 (4)	0.0613 (7)*	0.411 (6)
F6'	1.2569 (6)	0.8560 (12)	0.1386 (4)	0.0613 (7)*	0.411 (6)
C14A	1.1663 (3)	0.8216 (7)	0.0908 (2)	0.0589 (11)	0.179 (3)
F4A	1.1322 (12)	0.867 (2)	0.0240 (6)	0.0613 (7)*	0.179 (3)
F5A	1.2639 (10)	0.769 (2)	0.1061 (9)	0.0613 (7)*	0.179 (3)
F6A	1.1623 (13)	1.0042 (19)	0.1205 (9)	0.0613 (7)*	0.179 (3)

### Atomic displacement parameters (Å^2^)

**Table:**

	*U*^11^	*U*^22^	*U*^33^	*U*^12^	*U*^13^	*U*^23^
Ge1A	0.0323 (2)	0.03686 (19)	0.0365 (2)	0.00216 (19)	0.00519 (16)	0.0002 (2)
C15A	0.042 (3)	0.046 (7)	0.062 (3)	0.005 (3)	0.021 (3)	0.017 (4)
C16A	0.032 (3)	0.065 (4)	0.072 (7)	−0.005 (3)	0.006 (4)	0.022 (6)
C17A	0.042 (3)	0.052 (5)	0.045 (3)	−0.009 (4)	−0.002 (4)	0.003 (3)
Ge1	0.0323 (2)	0.03686 (19)	0.0365 (2)	0.00216 (19)	0.00519 (16)	0.0002 (2)
C15	0.042 (3)	0.046 (7)	0.062 (3)	0.005 (3)	0.021 (3)	0.017 (4)
C16	0.032 (3)	0.065 (4)	0.072 (7)	−0.005 (3)	0.006 (4)	0.022 (6)
C17	0.042 (3)	0.052 (5)	0.045 (3)	−0.009 (4)	−0.002 (4)	0.003 (3)
C1	0.0292 (19)	0.0364 (17)	0.034 (2)	−0.0018 (15)	0.0055 (16)	−0.0001 (16)
C2	0.048 (2)	0.045 (2)	0.037 (2)	0.0075 (18)	0.0078 (19)	−0.0067 (17)
C3	0.051 (2)	0.0427 (19)	0.047 (2)	0.0167 (19)	0.007 (2)	0.0012 (19)
C4	0.036 (2)	0.0454 (19)	0.039 (2)	0.0012 (17)	0.0077 (18)	0.0022 (18)
C5	0.049 (2)	0.051 (2)	0.035 (2)	0.0023 (19)	0.0048 (19)	−0.0080 (19)
C6	0.043 (2)	0.037 (2)	0.047 (2)	0.0069 (17)	0.0042 (19)	−0.0044 (18)
C8	0.037 (2)	0.0359 (17)	0.035 (2)	0.0027 (16)	0.0030 (17)	−0.0011 (16)
C9	0.039 (2)	0.048 (2)	0.050 (2)	0.0036 (18)	0.006 (2)	0.0091 (19)
C10	0.034 (2)	0.056 (2)	0.059 (3)	0.0020 (19)	0.007 (2)	0.006 (2)
C11	0.044 (2)	0.046 (2)	0.045 (2)	−0.0039 (18)	0.0148 (19)	−0.0005 (18)
C12	0.048 (2)	0.055 (3)	0.045 (2)	−0.004 (2)	0.006 (2)	0.0112 (19)
C13	0.035 (2)	0.057 (2)	0.041 (2)	−0.0023 (19)	−0.0019 (18)	0.0086 (19)
C7	0.058 (3)	0.063 (3)	0.041 (2)	0.009 (3)	0.004 (2)	0.006 (2)
F1	0.110 (8)	0.054 (3)	0.061 (6)	−0.009 (4)	−0.013 (4)	0.019 (3)
F2	0.110 (8)	0.089 (7)	0.042 (4)	0.048 (6)	0.034 (4)	0.022 (4)
F3	0.044 (5)	0.093 (6)	0.062 (5)	0.014 (4)	−0.011 (4)	0.014 (4)
C7A	0.058 (3)	0.063 (3)	0.041 (2)	0.009 (3)	0.004 (2)	0.006 (2)
F1A	0.110 (12)	0.108 (12)	0.075 (12)	−0.022 (10)	−0.012 (7)	0.050 (9)
F2A	0.078 (7)	0.099 (11)	0.023 (5)	0.012 (7)	0.001 (5)	0.015 (6)
F3A	0.103 (13)	0.122 (17)	0.053 (8)	0.080 (12)	0.029 (8)	0.013 (8)
C14	0.055 (3)	0.062 (3)	0.059 (3)	0.005 (2)	0.015 (2)	0.006 (2)
C14'	0.055 (3)	0.062 (3)	0.059 (3)	0.005 (2)	0.015 (2)	0.006 (2)
C14A	0.055 (3)	0.062 (3)	0.059 (3)	0.005 (2)	0.015 (2)	0.006 (2)

### Geometric parameters (Å, °)

**Table:**

Ge1A—C1	1.944 (3)	C3—C4	1.372 (5)
Ge1A—C8	1.947 (4)	C3—H3A	0.9500
Ge1A—C17A	1.961 (4)	C4—C5	1.382 (5)
Ge1A—C15A	1.969 (5)	C4—C7	1.486 (5)
Ge1A—C16A	2.463 (6)	C5—C6	1.381 (5)
C15A—C16A	1.562 (11)	C5—H5A	0.9500
C15A—H15C	0.9900	C6—H6A	0.9500
C15A—H15D	0.9900	C8—C13	1.384 (5)
C16A—C17A	1.591 (13)	C8—C9	1.389 (5)
C16A—H16C	0.9900	C9—C10	1.381 (5)
C16A—H16D	0.9900	C9—H9A	0.9500
C17A—H17C	0.9900	C10—C11	1.382 (5)
C17A—H17D	0.9900	C10—H10A	0.9500
C15—C16	1.563 (11)	C11—C12	1.379 (5)
C15—H15A	0.9900	C11—C14	1.500 (6)
C15—H15B	0.9900	C12—C13	1.376 (5)
C16—C17	1.591 (13)	C12—H12A	0.9500
C16—H16A	0.9900	C13—H13A	0.9500
C16—H16B	0.9900	C7—F2	1.326 (9)
C17—H17A	0.9900	C7—F3	1.331 (9)
C17—H17B	0.9900	C7—F1	1.342 (9)
C1—C6	1.383 (5)	C14—F5	1.302 (8)
C1—C2	1.385 (5)	C14—F6	1.340 (7)
C2—C3	1.381 (5)	C14—F4	1.363 (7)
C2—H2A	0.9500		
			
C1—Ge1A—C8	108.72 (15)	C3—C2—C1	121.0 (3)
C1—Ge1A—C17A	118.9 (11)	C3—C2—H2A	119.5
C8—Ge1A—C17A	118.7 (11)	C1—C2—H2A	119.5
C1—Ge1A—C15A	120.2 (12)	C4—C3—C2	120.4 (3)
C8—Ge1A—C15A	109.8 (11)	C4—C3—H3A	119.8
C17A—Ge1A—C15A	77.8 (3)	C2—C3—H3A	119.8
C1—Ge1A—C16A	120.4 (4)	C3—C4—C5	119.5 (3)
C8—Ge1A—C16A	130.5 (4)	C3—C4—C7	119.3 (3)
C16A—C15A—Ge1A	87.7 (4)	C5—C4—C7	121.2 (4)
C16A—C15A—H15C	114.0	C6—C5—C4	119.8 (3)
Ge1A—C15A—H15C	114.0	C6—C5—H5A	120.1
C16A—C15A—H15D	114.0	C4—C5—H5A	120.1
Ge1A—C15A—H15D	114.0	C5—C6—C1	121.4 (3)
H15C—C15A—H15D	111.2	C5—C6—H6A	119.3
C15A—C16A—C17A	103.0 (8)	C1—C6—H6A	119.3
C15A—C16A—Ge1A	53.0 (3)	C13—C8—C9	118.1 (4)
C17A—C16A—Ge1A	52.7 (2)	C13—C8—Ge1A	119.9 (3)
C15A—C16A—H16C	111.2	C9—C8—Ge1A	121.9 (3)
C17A—C16A—H16C	111.2	C10—C9—C8	120.9 (4)
Ge1A—C16A—H16C	139.4	C10—C9—H9A	119.5
C15A—C16A—H16D	111.2	C8—C9—H9A	119.5
C17A—C16A—H16D	111.2	C9—C10—C11	119.9 (4)
Ge1A—C16A—H16D	111.5	C9—C10—H10A	120.1
H16C—C16A—H16D	109.1	C11—C10—H10A	120.1
C16A—C17A—Ge1A	87.1 (5)	C12—C11—C10	119.8 (4)
C16A—C17A—H17C	114.1	C12—C11—C14	120.4 (4)
Ge1A—C17A—H17C	114.1	C10—C11—C14	119.8 (4)
C16A—C17A—H17D	114.1	C13—C12—C11	119.8 (4)
Ge1A—C17A—H17D	114.1	C13—C12—H12A	120.1
H17C—C17A—H17D	111.3	C11—C12—H12A	120.1
C16—C15—H15A	114.1	C12—C13—C8	121.4 (4)
C16—C15—H15B	114.1	C12—C13—H13A	119.3
H15A—C15—H15B	111.2	C8—C13—H13A	119.3
C15—C16—C17	102.6 (8)	F2—C7—F3	105.6 (9)
C15—C16—H16A	111.2	F2—C7—F1	108.0 (9)
C17—C16—H16A	111.2	F3—C7—F1	103.0 (9)
C15—C16—H16B	111.2	F2—C7—C4	117.0 (7)
C17—C16—H16B	111.2	F3—C7—C4	110.1 (8)
H16A—C16—H16B	109.2	F1—C7—C4	112.0 (7)
C16—C17—H17A	114.1	F5—C14—F6	125.9 (6)
C16—C17—H17B	114.1	F5—C14—F4	107.2 (5)
H17A—C17—H17B	111.3	F6—C14—F4	82.5 (6)
C6—C1—C2	117.9 (3)	F5—C14—C11	113.7 (5)
C6—C1—Ge1A	121.2 (3)	F6—C14—C11	111.6 (4)
C2—C1—Ge1A	120.8 (3)	F4—C14—C11	110.2 (4)
			
C1—Ge1A—C15A—C16A	−102.4 (14)	Ge1A—C1—C6—C5	179.0 (3)
C8—Ge1A—C15A—C16A	130.5 (14)	C1—Ge1A—C8—C13	−170.8 (3)
C17A—Ge1A—C15A—C16A	14 (2)	C17A—Ge1A—C8—C13	49.1 (6)
Ge1A—C15A—C16A—C17A	−18 (2)	C15A—Ge1A—C8—C13	−37.5 (9)
C1—Ge1A—C16A—C15A	102 (2)	C16A—Ge1A—C8—C13	1.8 (7)
C8—Ge1A—C16A—C15A	−70 (2)	C1—Ge1A—C8—C9	6.4 (4)
C17A—Ge1A—C16A—C15A	−158 (3)	C17A—Ge1A—C8—C9	−133.7 (6)
C1—Ge1A—C16A—C17A	−100.0 (19)	C15A—Ge1A—C8—C9	139.7 (9)
C8—Ge1A—C16A—C17A	88.2 (19)	C16A—Ge1A—C8—C9	179.0 (7)
C15A—Ge1A—C16A—C17A	158 (3)	C13—C8—C9—C10	0.7 (6)
C15A—C16A—C17A—Ge1A	18 (2)	Ge1A—C8—C9—C10	−176.5 (3)
C1—Ge1A—C17A—C16A	104.0 (13)	C8—C9—C10—C11	0.4 (6)
C8—Ge1A—C17A—C16A	−119.9 (14)	C9—C10—C11—C12	−0.9 (6)
C15A—Ge1A—C17A—C16A	−13.9 (19)	C9—C10—C11—C14	178.0 (4)
C8—Ge1A—C1—C6	86.7 (3)	C10—C11—C12—C13	0.3 (6)
C17A—Ge1A—C1—C6	−133.3 (7)	C14—C11—C12—C13	−178.6 (4)
C15A—Ge1A—C1—C6	−40.9 (8)	C11—C12—C13—C8	0.9 (6)
C16A—Ge1A—C1—C6	−86.8 (6)	C9—C8—C13—C12	−1.4 (6)
C8—Ge1A—C1—C2	−94.4 (3)	Ge1A—C8—C13—C12	175.9 (3)
C17A—Ge1A—C1—C2	45.6 (7)	C3—C4—C7—F2	−167.6 (9)
C15A—Ge1A—C1—C2	138.0 (8)	C5—C4—C7—F2	12.3 (10)
C16A—Ge1A—C1—C2	92.1 (6)	C3—C4—C7—F3	71.9 (9)
C6—C1—C2—C3	0.0 (6)	C5—C4—C7—F3	−108.2 (9)
Ge1A—C1—C2—C3	−178.9 (3)	C3—C4—C7—F1	−42.2 (8)
C1—C2—C3—C4	0.0 (6)	C5—C4—C7—F1	137.8 (7)
C2—C3—C4—C5	−0.1 (6)	C12—C11—C14—F5	−82.7 (6)
C2—C3—C4—C7	179.9 (4)	C10—C11—C14—F5	98.4 (6)
C3—C4—C5—C6	0.2 (6)	C12—C11—C14—F6	127.5 (6)
C7—C4—C5—C6	−179.8 (4)	C10—C11—C14—F6	−51.4 (7)
C4—C5—C6—C1	−0.2 (6)	C12—C11—C14—F4	37.7 (7)
C2—C1—C6—C5	0.1 (6)	C10—C11—C14—F4	−141.2 (6)
